# Crystal Structures and Intermolecular Interactions in α‐ and β‐phosgene

**DOI:** 10.1002/anie.202517323

**Published:** 2025-11-14

**Authors:** Sven Ringelband, Jonathan Pfeiffer, A. Dominic Fortes, Christopher M. Howard, Stewart F. Parker, Antti J. Karttunen, Frank Tambornino

**Affiliations:** ^1^ Institute of Chemistry University Marburg Hans‐Meerwein‐Str. 4 35043 Marburg Germany; ^2^ ISIS Neutron and Muon Source STFC Rutherford Appleton Laboratory Chilton OX11 0QX United Kingdom; ^3^ Department of Chemistry and Materials Science Aalto University Kemistintie 1 Espoo 02150 Finland

**Keywords:** Crystallography, DFT calculations, Inelastic neutron scattering, Neutron diffraction, Phosgene

## Abstract

The crystal structures of α‐ and β‐phosgene have been elucidated on the basis of variable temperature neutron powder diffraction. Although the stable α‐phase closely mirrors the published structure ($I4_{1}/a$, No. 88), the new metastable β‐phase crystallises in $Cmc2_{1}$ (No. 36, at 135 K: *a* = 10.244042(22) Å, *b* = 6.280321(21) Å, *c* = 5.46069(4) Å). Both modifications show dipole–dipole interactions, but only α‐phosgene shows pronounced σ‐hole interactions. Quantum chemical calculations in both solid state and of model dimers in gas‐phase reveal a complex interplay of intermolecular interactions. The first inelastic neutron scattering spectra of α‐phosgene shows an unusual separation of the translational and librational modes, alongside sharp bands for the internal modes. Our computational studies also show an unusual, extensive mixing of the in‐phase C–Cl stretch ($\nu_{2}$) and the out‐of‐plane bend ($\nu_{6}$) modes, which are symmetry‐forbidden from mixing in the gas‐phase.

## Graphical abstract

Despite its notorious reputation as a war gas, phosgene (${\mathrm{COCl}}_{2}$) is one of the most important intermediate chemicals in industry. In this study, we present the crystal structures of two phosgene polymorphs as deduced from powder neutron diffraction and show which intermolecular interactions stabilise the different modifications. The first inelastic neutron scattering spectra show an unusual separation of the translational and librational modes, alongside sharp bands for the internal modes.

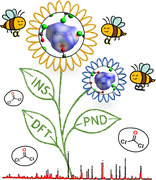

## Introduction

It was in 1812 that Sir Humphrey Davy's younger brother, John Davy, first discovered phosgene.^[^ ^1^ ^]^ Formally trained in medicine, he was an amateur chemist and among his many discoveries, the seminal synthesis of phosgene has proven to be his most impactful in the long run. In his work, he notes that Gay Lussac, Thénard, and Murray have previously exposed a mixture of carbon monoxide and chlorine to light, but failed to observe any reaction.^[^ ^2^ ^]^ The professional discord between the latter and Humphrey Davy prompted John Davy to repeat the experiment. In contrast to his contemporaries, he found the reaction to proceed rapidly with loss of the typical chlorine colour and formation of a reduced pressure in the sealed flask.

The thus‐synthesised gas was distinctly different from all hitherto known substances and Davy describes a multitude of reactions that are typical for phosgene. For example: It reacts slowly with water generating carbonic and hydrochloric acid, but reacts rapidly under generation of heat with ammonia.^[^ ^1^ ^]^ From there on, phosgene was mostly a laboratory curiosity, but its potential use as viable industrial starting material led to the development of a catalytic synthetic process.^[^ ^3^ ^]^ From the 1880s onward, phosgene was used for the synthesis of fine chemicals, mostly dyes, and its pre‐World War I production volume was ca. 5000 metric tons per year.^[^ ^4^ ^]^

In his seminal paper, John Davy described the smell of phosgene as ‘more intolerable and suffocating than chlorine itself.'^[^ ^1^ ^]^ Its high toxicity, combined with ease of handling, later made it a weapon of choice in gas warfare during World War I. Even low to moderate exposure can cause a range of symptoms, including mucous membrane irritation, coughing, dizziness, chest tightness, and shallow breathing.^[^ ^5^ ^]^ Although these symptoms often subside quickly once exposure ends, they are typically followed by a deceptively symptom‐free latent phase that may last up to 24 h. As this phase wanes, respiratory distress can escalate, potentially culminating in death from respiratory or cardiac failure. With immediate and sustained medical care, however, full recovery without lasting damage is possible. High‐dosage exposures on the other hand lead to death by heart failure within minutes.^[^ ^5^ ^]^

Despite its toxicity, phosgene is nowadays used as an important starting material. It ranks among the most important industrial chemicals and serves as a central building block for agrochemicals, polycarbonates, pharmaceuticals, dyes, and other fine chemicals.^[^ ^4^ ^]^ In addition to traditional synthetic routes based on the direct reaction of CO and ${\mathrm{Cl}}_{2}$, alternative approaches have recently been reported that employ chloride‐based reagents to activate molecular chlorine under mild conditions, allowing for phosgene generation at ambient temperature and pressure.^[^ ^6^ ^]^ Owing to its broad industrial relevance, the physicochemical properties of phosgene have been thoroughly investigated. Under ambient conditions, it is a colourless gas with a density approximately 3.5 times that of air. At 280 K, it condenses into a mobile, colourless liquid and freezes at 145.35 K to give a crystalline solid. Although the gas and liquid phases have been extensively studied, the solid state has received comparatively little attention.^[^ ^4^, ^7^ ^]^

In chemistry, polymorphism means that a compound can adopt different crystal structures. The discovery of this phenomenon dates back to 1788, where Klaphroth found the three polymorphic forms of calcium carbonate.^[^ ^8^ ^]^ Wöhler and Liebig found the first instance of polymorphism for a molecular compound: benzamide.^[^ ^9^ ^]^ However, it was not until 1938 that structures of a polymorphic compound were elucidated by X‐ray diffraction on the example of resorcinol.^[^ ^10^, ^11^ ^]^ A more recent review found that polymorphism occurs in at least half of all molecular compounds and as such is far from being a rare phenomenon.^[^ ^12^ ^]^ For phosgene, initial calorimetric measurements indicated residual entropy in the solid, suggesting structural disorder.^[^ ^13^ ^]^ However, single‐crystal data published in 1952 revealed an ordered crystal structure, hereafter called α‐phosgene.^[^ ^14^ ^]^ Subsequent thermodynamic studies established the existence of three polymorphs,^[^ ^15^ ^]^ with the early calorimetric data likely corresponding to a metastable form. However, the crystal structures of the metastable solids (originally named solid II, and solid III, resp.) have not been investigated.

In this work, we present a thorough crystallographic characterisation of solid phosgene, in which we disseminate the crystal structure of metastable β‐phosgene (formerly called solid II) for the first time. The differences in molecular packing are highlighted by Hirshfeld surface analysis, which indicates that a σ‐hole interaction is present in the stable α‐phase but not in the β‐phase. High‐level quantum chemical calculations show that a combination of different van der Waals interactions are responsible for the stability of the structures, which show only 0.5 kJ ${\mathrm{mol}}^{-1}$ energy difference per formula unit. The first reported inelastic neutron scattering spectra of α‐phosgene shows some unusual behaviour.

## Results and Discussion

### Growth of a Sample of β‐Phosgene and Its Structure Solution

Based on the aforementioned previous reports, we attempted the crystallisation of β‐phosgene using the single‐crystal diffractometers available at our institution. A small sample of commercially available phosgene was degassed and condensed into a glass capillary, which was then flame‐sealed under its own vapor pressure at 273 K. The capillary was mounted on the goniometer of a single‐crystal X‐ray diffractometer at room temperature and gradually cooled in a nitrogen cryostream. Upon approaching the reported melting point of phosgene (145.35 K), no crystallisation was observed. Instead, the sample supercooled to approximately 130 K, where it rapidly solidified into an opaque mass. Initial measurements revealed only the characteristic scattering pattern of a poorly crystalline polycrystalline material. This behaviour was consistently reproduced in repeated experiments.

Careful heating close to the melting temperature resulted in partial melting of the sample and through Ostwald‐ripening a crystal was grown from melt. Test measurements confirmed the presence of a single specimen exhibiting lattice parameters consistent with the previously reported structure by Lipscomb.^[^ ^14^ ^]^ The crystal was subsequently cooled to 80 K and a full dataset was collected, which corroborated the structure from literature within close margins; details are discussed below.

Clearly, we observe supercooling of liquid phosgene and its subsequent solidification in a capillary with the temperatures similar to those observed by Ott, however, the growth of a single crystal of the metastable form proved impossible for us.^[^ ^13^, ^15^ ^]^ Consequently, we pursued structure determination of β‐phosgene *via* powder X‐ray diffraction. A new capillary sample, analogous to the single‐crystal setup, was mounted on our in‐house powder diffractometer. Upon cooling, the sample again supercooled and rapidly crystallised around 130 K, but poor crystallinity rendered the diffraction data unusable.

To allow for a precise temperature control and deal with possibly poor crystallinity, we chose to use neutron powder diffraction to elucidate the crystal structure of β‐phosgene. At ISIS Neutron and Muon Source, a fresh sample of phosgene was prepared from triphosgene following the literature procedure.^[^ ^16^ ^]^ Approximately 5 mL of phosgene was condensed into an aluminum slab‐geometry sample holder, filled with pre‐dried glass wool to minimize preferred orientation and suppress single‐crystal domains.

**Figure 1:**
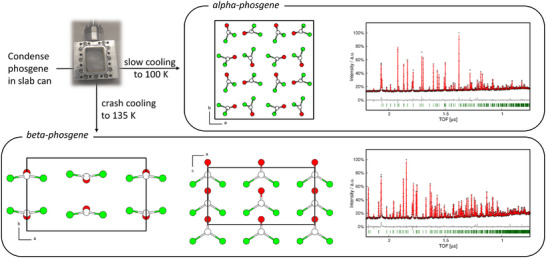
Top left: Workflow for the formation of pure samples of α‐ and β‐phosgene; the open slab can filled with glass wool is shown as photograph. Top right: Crystal structure and Rietveld plot of α‐phosgene at 100 K. Bottom: Crystal structure and Rietveld plot of β‐phosgene at 135 K. Colour code: C white, O red, Cl green. Atoms are drawn with arbitrary radii. Rietveld: Black crosses display measured data, the red line represents the refined model, green bars indicate Bragg positions and the grey line displays the difference plot.

An initial measurement at 100 K was performed, which showed the diffraction pattern expected for α‐phosgene and data suitable for a Rietveld refinement was collected, see Figure 1 top. Measurements in 10 K increments down to a temperature of 12 K all showed a similar diffraction pattern and the full Rietveld refinement of the data collected at 12 K yielded a model for α‐phosgene close to the thermal limit.

We then attempted to grow β‐phosgene by following a reported thermal protocol.^[^ ^15^ ^]^ The sample was heated above its melting point to 170 K. Next, the sample was cooled with the maximum rate (3.75 K $\min^{-1}$) from 170 to 140 K to produce a supercooled melt evident by the missing response of the heat of crystallisation on the thermocouple and, more importantly, by the absence of sharp Bragg peaks. Subsequently, a very slow ramp of 0.0167 K $\min^{-1}$ was applied to cool to 135 K during which time β‐phosgene appeared. After 3 h 14 m, at a temperature of 136.8 K, the sample spontaneously crystallised, concomitant with a small spike (0.2 K, duration 90 s) in the observed temperature. Data for this metastable phase (β‐phosgene) was collected for 3 h 50 m to allow for indexing, structure solution and subsequent Rietveld refinement, yielding the diffraction pattern shown in Figure 1 bottom. This pattern clearly differs from that of α‐phosgene. Indexing indicated orthorhombic symmetry, with systematic absences compatible with space groups $Cmc2_{1}$ (non‐centrosymmetric) and Cmce (centrosymmetric). Structure solution *via* charge flipping in $Cmc2_{1}$ followed by symmetry analysis with PLATON (ADDSYM) indicated no missed symmetry elements.^[^ ^17^ ^]^ Expansion of the model to P1 followed by Rietveld refinement and further symmetry checks unambiguously confirmed $Cmc2_{1}$ as the correct space group.

### Crystal Structures of α‐ and β‐Phosgene and Their Hirshfeld Surface Analysis

α‐Phosgene crystallises in the tetragonal system with space group $I4_{1}/a$ (No. 88, SC‐XRD at 80 K: a=15.6974(4) Å, c=5.6780(2) Å; powder neutron diffraction at 100 K: a=15.71613(4) Å, c=5.684833(25) Å; powder neutron diffraction at 12 K: a=15.58245(4) Å, c=5.655737(25) Å), 16 molecules per unit cell (Z=16) and one crystallographically independent molecule in the asymmetric unit. All atoms occupy the general position with Wyckoff number 16*f*. The molecule adopts a geometry close to the ideal $C_{2v}$ point group; the ideal shape is not found probably due to crystal packing effects. For the corresponding factor group analysis, see below in the chapter on inelastic neutron scattering. At 100 K the C–O distance is 1.1842(23) Å and the C–Cl distances are 1.7175(24) Å and 1.7317(21) Å (at 80 K: C–O: 1.1748(26) Å, C–Cl1: 1.7282(18) Å, C–Cl2: 1.7394(2) Å, at 12K: C–O: 1.1833(18) Å, C–Cl1: 1.7203(17) Å, C–Cl2: 1.7471(14) Å). These values closely mirror the model from literature.^[^ ^14^ ^]^

β‐Phosgene crystallises in the orthorhombic system with the non‐centrosymmetric space group $Cmc2_{1}$ (No. 36, at 135 K: a=10.244042(22) Å, b=6.280321(21) Å, c=5.46069(4) Å), four molecules in the unit cell (Z=4) and one crystallographically independent molecule in the asymmetric unit. C1 and O1 occupy Wyckoff position 4*a*, whereas Cl1 occupies the general position with Wyckoff letter 8*b*. The molecule adopts a geometry close to the ideal $C_{2v}$ point group with a deviation somewhat larger than in α‐phosgene. Although the C–Cl distance and O–C–Cl angles are similar due to space group symmetry, a torsion angle of ca. 3.4(3) between Cl–C–Cl / C–O is present. At 135 K the C–O distance (1.219(5) Å) is slightly larger, and the C–Cl distance (1.7029(25) Å) is slightly shorter than in the α‐polymorph, hinting at the fact that different intermolecular forces are at play stabilising the respective structures.

In the extended structure the molecules in β‐phosgene are arranged in sheets that lie in the (101) plane. Individually, the sheets form a motif of the hexagonal packing, which deviates only slightly from its ideal form (118.05). The neighbouring sheets are shifted with respect to the first by $\frac{1}{2}a$ and $\frac{1}{2}c$; the packing motif of the α‐U‐type results.^[^ ^18^ ^]^ The sheets themselves are puckered: Molecules alternate in pointing the CO bond slightly along b and −b. Additionally, the topology of the underlying nets that are constructed from the centroids of the phosgene molecules can be analysed, which has been performed with TOPOS Pro.^[^ ^19^ ^]^ In both polymorphs, the molecules are surrounded by 14 other molecules. For α‐phosgene, the topology of 14T12 results, which is a distorted variant of the bcu‐x net. In contrast, for β‐phosgene we find the non‐distorted bcu‐x topology.

The only other reported carbonyl halide crystal structure is that of thiophosgene.^[^ ^20^ ^]^ However, its structure bears neither resemblance to α‐ nor to β‐phosgene. A comparison to oxalyl chloride and its intermolecular interactions might prove useful; however, the published crystal structure contains some uncertainties, making a meaningful comparison complicated.^[^ ^21^ ^]^

**Figure 2:**
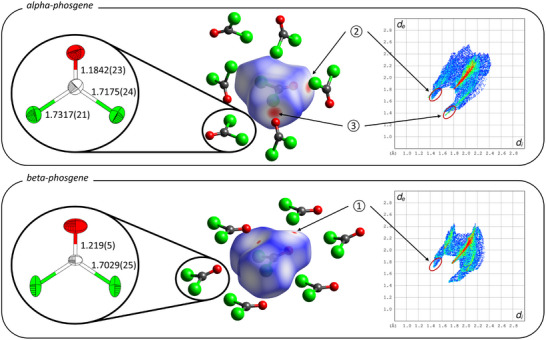
Left: Molecular structure of phosgene in the crystal structures of α‐ (top) and β‐phosgene (bottom), determined by neutron powder diffraction at 100 and 135 K, respectively. Ellipsoids at 75% probability and bond lenghts given in Å. Middle: Excerpts of α‐ and β‐phosgene structures (top, bottom) with corresponding Hirshfeld surfaces; red areas indicate intermolecular contacts shorter than the sum of van der Waals radii. Right: Fingerprint plots for α‐ and β‐phosgene (top, bottom), with regions corresponding to red Hirshfeld surface areas highlighted. (1) and (2): C⋯O contacts; (3): O⋯Cl contacts. Colour code: C white, O red, Cl green.

Hirshfeld surface analysis is uniquely suitable to understand the deformation of molecular space in the crystalline environment. As a measure of the space a molecule occupies in the crystal, this surface captures the entirety of its intermolecular interactions and contacts. Figure 2 middle shows the respective Hirshfeld surfaces for α‐ and β‐phosgene, respectively, including relevant intermolecular contacts. For β‐phosgene, the Hirshfeld surface shows two distances external to the surface that are closer than the van der Waals radii of the involved elements and these areas are marked red. All other contacts are either similar (white) or further apart than the sum of the van der Waal radii, indicating low degrees of attractive intermolecular interactions. For β‐phosgene, both interactions correspond to the dipole–dipole interaction of the oxygen atom of a C═O group, which is negatively polarized, with a carbon atom of an adjacent molecule, which is positively polarised. In the fingerprint plot (see Figure 2 bottom right) this corresponds to the ‘spike’ as seen at $d_{e}$/$d_{i}$ values of 1.7/1.5 and 1.5/1.7 Å. This attractive interaction is the reason why the molecules do not lie flat in the (101) plane, but instead form wavy sheets (see Figure 1 bottom left), deviating from the possible aristotype in space group Fmm2 (No. 42).

In contrast, the Hirshfeld surface of α‐phosgene reveals two distinct types of attractive intermolecular interactions. The first is, once again, a dipole–dipole interaction between the oxygen atom of a C═O group and the carbon atom of a neighbouring molecule. This interaction is marked as ‘2’ in the top right panel of Figure 2, and the corresponding spike in the fingerprint plot is highlighted by a red ellipse. The second interaction is of a different nature. Here, the oxygen atom of a C═O group lies adjacent to a chlorine atom from a neighbouring molecule, with the resulting C═O···Cl angle approaching linearity (160.73(7)). This electrostatic interaction arises from the region of positive electrostatic potential extending along the C─Cl bond into which electron density is donated by the oxygen atom and constitutes a prime example of a σ‐hole interaction. Due to the donation of electron density into the σ* orbital of the C─Cl bond, it is about 0.03 Å longer than in β‐phosgene (α‐phosgene: 1.7175(24) and 1.7317(21) Å, β‐phosgene: 1.7029(25) Å), which is a typical value for these interactions.^[^ ^22^ ^]^ In the fingerprint plot, this interaction also forms a spike, which is marked with ‘3’ in the top right panel of Figure 2 and is in close proximity to that of the dipole–dipole interactions. The breakdown of the fingerprint plot into the contributions from the individual atom types can be found in the Supporting Information.

This kind of Hirshfeld surface analysis, however, is largely phenomenological. More precisely, it cannot associate concrete energies to the different interactions and so it remains an open question, whether the dipole–dipole interactions or the σ‐hole interactions dominate.

### Quantum Chemical Calculations

In order to fully understand the phase transitions and the intermolecular interactions in α‐ and β‐phosgene, we performed quantum chemical calculations both for the crystal structure as well as for model systems in the gas phase. The solid‐state calculations on the energetics and thermodynamics of both phosgene polymorphs were carried out with dispersion‐corrected hybrid density functional methods, using DFT‐PBE0‐D3(BJ+ABC)/def2‐TZVP level of theory and the CRYSTAL program package (full computational details are available in Supporting Information).^[^ ^23^, ^24^, ^25^, ^26^, ^27^ ^]^

We first fully optimised the crystal structures of α‐ and β‐phosgene with DFT (at 0 K). In molecular crystals like phosgene, accounting for weak van der Waals interactions is crucial. By using the dispersion‐corrected DFT‐PBE0‐D3(BJ+ABC) method and unmodified molecular def2‐TZVP basis set, we obtained good agreement with experimentally determined crystal structures. For α‐phosgene, the optimised lattice parameters are (differences to T=12 K crystal structure in parentheses): a=15.72 Å (+0.9%) and c=5.73 Å (+1.4%). For β‐phosgene, the lattice parameters are (differences to T=135 K crystal structure in parentheses): a=10.16 Å (−0.8%), b=6.16 Å (−1.8%), and c=5.33 Å (+1.2%). Optimized C═O distance is 1.18 Å in both polymorphs. C–Cl distances are 1.72–1.74 Å in α‐phosgene and 1.73 Å in β phosgene.

After optimising the crystal structures, we investigated the energetics and thermodynamics of the polymorphs. When comparing electronic energies at 0 K, the energy difference is very small: α‐phosgene is 0.5 kJ ${\mathrm{mol}}^{-1}$ per formula unit lower in energy compared to β‐phosgene. We also compared the 0 K lattice energies, defined as the energy difference between the crystalline solid (per formula unit) and a phosgene molecule in the gas phase. The 0 K lattice energies of α‐ and β‐phosgene are 37.8 and 37.3 kJ ${\mathrm{mol}}^{-1}$ per formula unit, respectively.

We also investigated the Gibbs free energies of the polymorphs at several temperatures to get a better understanding of the role of the temperature. For this, the harmonic frequencies of the crystalline solids were calculated and compared (using a 2×2×1 phonon supercell for β‐phosgene to have the same number of atoms as in α‐phosgene). We calculated the entropy and the Gibbs free energy for the phosgene polymorphs at several temperatures between 10 and 150 K, and the entropy term is larger in β‐phosgene throughout the temperature range. For example, at T=100 K, entropies of the α‐ and β‐polymorphs are 55.9 and 59.4 J (mol K)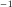 per formula unit, respectively. At T=10 K, the Gibbs free energy of α‐phosgene is 0.4 kJ ${\mathrm{mol}}^{-1}$ per formula unit lower compared to β‐phosgene. At T=100 K, the α‐polymorph is favored only by 0.1 kJ ${\mathrm{mol}}^{-1}$ per formula unit. Finally, at about 130 K, the Gibbs free energies of the polymorphs are practically identical, and at T=150 K, close to the melting point of 145.35 K, β‐phosgene would be 0.1 kJ ${\mathrm{mol}}^{-1}$ per formula unit lower in Gibbs free energy compared to α‐phosgene. It should be noted that the Gibbs free energy comparisons are based only on harmonic frequency calculations and anharmonic effects are neglected.

**Figure 3:**
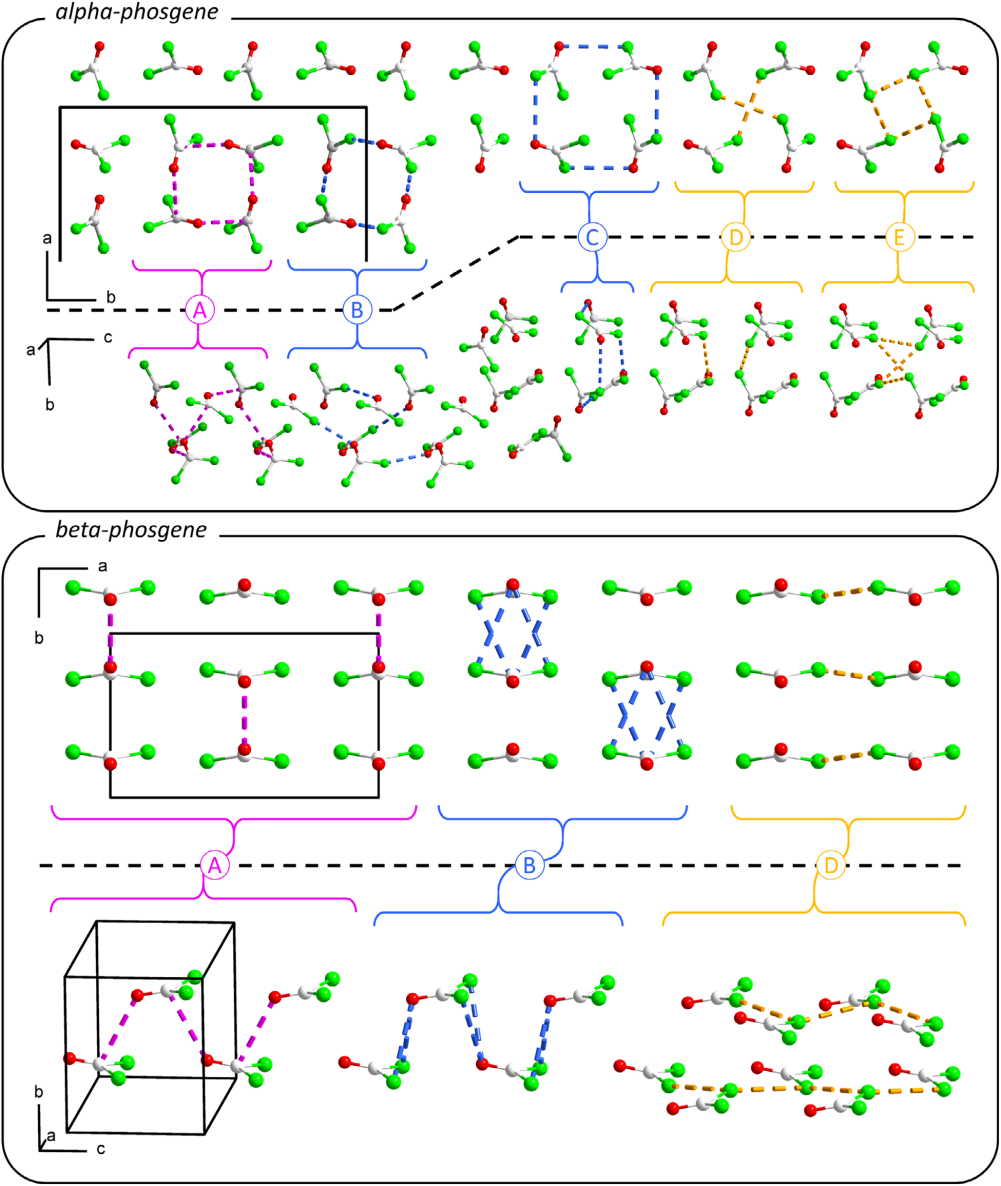
Overview of the different intermolecular interactions in α‐phosgene and β‐phosgene. Each part of the figure shows two different orientations of the respective crystal structures. Curly brackets indicate dimers A–E (for α‐phosgene) and dimers A–C (for β‐phosgene) as calculated from gas‐phase models, see text. Short intermolecular contacts are shown as dashed lines: C⋯O magenta, O⋯Cl blue, Cl⋯Cl orange. Color code: C white, O red, Cl green.

To investigate the intermolecular interactions in detail, we carried out molecular calculations on gas‐phase model systems using CCSD(F12*)(T*)/cc‐pVTZ‐F12 level of theory and the TURBOMOLE code (full computational details are available in Supporting Information).^[^ ^28^, ^29^, ^30^, ^31^, ^32^ ^]^ The gas‐phase model systems were used to compare intermolecular interactions between pairs of phosgene molecules extracted from the DFT‐optimised crystal structure. The studied phosgene dimers extracted from the α‐ and β‐phosgene are illustrated in Figure 3 (shown as contacts in the periodic crystal structures) and the intermolecular interaction energies obtained for each dimer are listed in Table 1. The studied dimers correspond to the nearest neighbours in the crystal lattice and Table 1 also lists the shortest intermolecular contacts for the studied dimers.

**Table 1:** Intermolecular interaction energies of shortest contacts in α‐ and β‐phosgene as modelled in gas‐phase model systems.

	$E_{int}$			Distance
System	(kJ ${\mathrm{mol}}^{-1}$)^a)^	Mult^b)^	Contact^c)^	(Å)
α‐phosgene				
Dimer A	−10.4	2	O⋯C	3.07
			O⋯O	3.38
			O⋯Cl	3.38
			O⋯Cl	3.53
Dimer B	−6.1	2	O⋯Cl	3.09
Dimer C	−9.1	2	O⋯Cl	3.52
			C⋯Cl	3.58
			O⋯Cl	3.83
			Cl⋯Cl	3.83
Dimer D	−3.0	1	Cl⋯Cl	3.62
Dimer E	−3.7	2	Cl⋯Cl	3.70
β‐phosgene				
Dimer A	−8.9	2	O⋯C	3.26
			O⋯Cl	3.41
			O⋯O	3.81
			Cl⋯C	3.97
Dimer B	−9.3	2	O⋯Cl	3.52
			O⋯C	3.70
			Cl⋯C	3.74
			Cl⋯Cl	3.90
Dimer C	−4.5	4	Cl⋯Cl	3.55
			O⋯Cl	3.70

^a)^ Interaction energy per dimer at the CCSD(F12*)(T*)/cc‐pVTZ‐F12 level of theory (in the gas phase).

^b)^ Number of pairwise interactions of this type for each molecule.

^c)^ Atom–atom distances shorter than 4 Å for the dimer.

The gas‐phase dimer calculations were carried out at a high level of theory to get an understanding of the relative strength of the two‐body intermolecular interactions in each phosgene polymorph. The calculated interaction energies between the dimers do not add up to the total lattice energy because three‐body and four‐body interactions have not been considered.^[^ ^33^ ^]^ Furthermore, the gas‐phase calculations were carried out at 0 K and thermal effects or vibrational contributions have not been considered. For both polymorphs, intermolecular O⋯C contacts are among the shortest ones and each phosgene molecule has two pairwise interactions of this type (dimer A). In the case of α‐phosgene, the dimer A shows the largest interaction energy. Even though there is almost equally short O⋯Cl distance in dimer B, the interaction energy for this dimer is smaller since the other interactomic contacts within this dimer are longer than 4 Å. Dimer C in α‐phosgene and dimer B in β‐phosgene both show O⋯Cl contact of 3.52 Å, together with three other contacts shorter than 4 Å. The interaction energy of these dimers is of similar magnitude in both polymorphs. Dimers D and E in α‐phosgene arise from longer Cl⋯Cl contacts with smaller intermolecular interaction energy. In β‐phosgene, the Cl⋯Cl contacts are somewhat shorter (dimer C) and the interaction energy is slightly larger compared to α‐phosgene. Overall, the phosgene polymorphs show different structural hierarchy and magnitudes of intermolecular interactions. There isn't any clear definitive difference between the polymorphs that makes α‐phosgene energetically and thermodynamically favourable at low temperatures.

### Inelastic Neutron Scattering

Gas electron diffraction^[^ ^34^ ^]^ and microwave spectroscopy^[^ ^34^ ^]^ have shown that phosgene has $C_{2v}$ symmetry in the gas phase. Phosgene has been extensively studied in the gas phase and the vibrational assignments are well‐established (ref. [4] provides a comprehensive review of the spectra). The six modes are composed of three totally symmetric in‐plane $A_{1}$ modes ($\nu_{1}$ carbonyl stretch at 1827 ${\mathrm{cm}}^{-1}$, $\nu_{2}$ in‐phase C–Cl stretch at 570 ${\mathrm{cm}}^{-1}$ and $\nu_{3}$ in‐phase Cl–C–Cl bend at 300 ${\mathrm{cm}}^{-1}$), two in‐plane $B_{1}$ modes ($\nu_{4}$ out‐of‐phase C–Cl stretch at 850 ${\mathrm{cm}}^{-1}$ and $\nu_{5}$ out‐of‐phase Cl–C–Cl bend at 440 ${\mathrm{cm}}^{-1}$) and an out‐of‐plane $B_{2}$ mode ($\nu_{6}$ the carbon atom moves perpendicular to the molecular plane at 585 ${\mathrm{cm}}^{-1}$). As far as we are aware, there are no vibrational spectroscopy studies of phosgene in the solid state.

**Figure 4:**
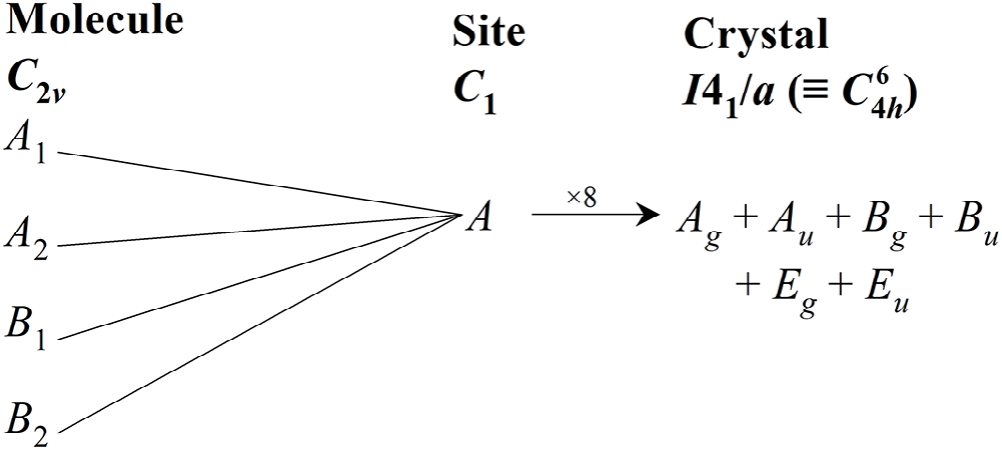
Correlation diagram for phosgene from the gas phase to the α‐polymorph.

The low temperature stable phase of phosgene crystallises in the tetragonal space group $I4_{1}/a$ ($C_{4h}^{6}$, No. 88) with eight molecules in the primitive cell, each occupying a site of $C_{1}$ symmetry. Figure 4 shows the correlation diagram.^[^ ^35^ ^]^ It can be seen that each gas phase mode gives rise to six (the *E* 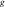 and *E* 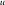 modes are doubly degenerate) modes in the tetragonal phase. In addition to the internal modes, the eight molecules give rise to 24 translational modes (3 acoustic and 21 optical modes) and 24 librational modes.

**Figure 5:**
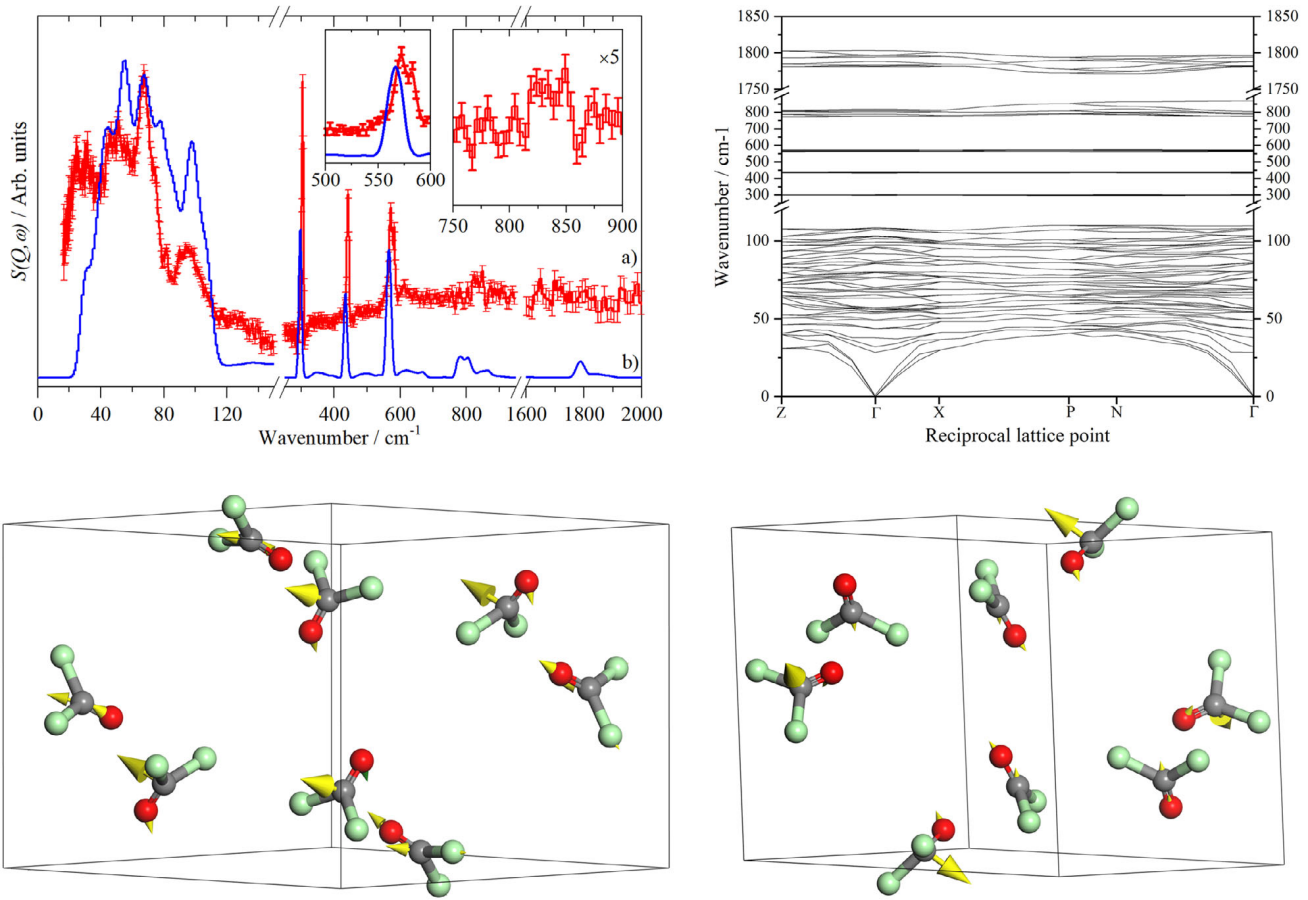
Top left: INS spectra of α‐phosgene: a) INS at 10 K and b) generated from a CASTEP calculation. The boxed inset is an abscissa expanded view of the 500–600 ${\mathrm{cm}}^{-1}$ region of $\nu_{2}$ and $\nu_{6}$. Two peaks are seen in the INS and CASTEP predicts these peaks to be within 7 ${\mathrm{cm}}^{-1}$ of each other so they are not resolved. The second inset is a x5 ordinate expansion of the 700–900 ${\mathrm{cm}}^{-1}$ region showing $\nu_{4}$ (out‐of‐phase C–Cl stretch). Top right: Calculated dispersion curves of α‐phosgene. Bottom: Examples of phosgene modes in the solid state that are nominally $\nu_{2}$ (bottom left) and $\nu_{6}$ (bottom right), respectively, it can be seen that both have molecules that are executing in‐plane ($\nu_{2}$‐type) and out‐of‐plane ($\nu_{6}$‐type) motions. Colour code: C gray, O red, Cl green.

Inelastic neutron scattering (INS) spectroscopy is a complementary form of vibrational spectroscopy. It has several distinct advantages for spectroscopy of the solid state, for the present work the most important are that there are no selection rules and that the low energy external modes are readily observable.^[^ ^36^ ^]^ Figure 5a shows the experimental INS spectrum. It can be seen that the translations and librations form a dense feature below 120 ${\mathrm{cm}}^{-1}$, $\nu_{3}$ and $\nu_{5}$ are clearly seen as sharp modes at 303 and 441 ${\mathrm{cm}}^{-1}$, $\nu_{2}$ and $\nu_{6}$ are very close together at 572 and 582 ${\mathrm{cm}}^{-1}$, $\nu_{4}$ is present as a weak, broad (50 ${\mathrm{cm}}^{-1}$, the resolution of TOSCA^[^ ^37^ ^]^ at this energy is 12 ${\mathrm{cm}}^{-1}$) band at 836 ${\mathrm{cm}}^{-1}$ and $\nu_{1}$ is below the detection limit. $\nu_{1}$ is dominated by motion of the C and O atoms, both of which have small total scattering cross sections (5.55 and 4.23 barn, 1 barn = $10^{-28}m^{2}$). In contrast, all of the other modes involve significant motion of the Cl atoms, of which 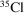 has a much larger cross section (21.8 barn). In addition, the high energy of the carbonyl stretch also means that it is more damped by the Debye–Waller factor.

Density functional theory calculations using CASTEP support the assignments and provide some further insight. Table S3 gives a complete list of the calculated modes at the Brillouin zone Γ‐point and their assignments. Also listed are the average of the factor group components and the difference between the highest and lowest energy of the factor group components.

Figure 5 (top left) shows the INS spectrum generated from a CASTEP calculation of the entire Brillouin zone and this is in reasonable agreement with the experimental spectrum. The mode visualisations confirm the assignments. In the external mode region, the translations occur below 70 ${\mathrm{cm}}^{-1}$ and the librations are the sharp peak at 82 ${\mathrm{cm}}^{-1}$ and the broad peak at 94 ${\mathrm{cm}}^{-1}$.

The calculated dispersion curves for phosgene are shown in Figure 5 top right. Those in the external mode region below 120 ${\mathrm{cm}}^{-1}$ (except for the acoustic translations) are almost flat, showing little vibrational dispersion (variation of transition energy with wavevector). This accounts for the unusual separation of the external modes into translations and librations. More often, because the transition energies of the translational and librational modes are similar and that they have common symmetry species, extensive mixing of the modes occurs.

Similarly, $\nu_{3}$, $\nu_{5}$, $\nu_{2}$, and $\nu_{6}$ are also flat, which accounts for the sharp bands. However, the mode visualisations show a marked surprise for $\nu_{2}$ and $\nu_{6}$: they are extensively mixed. In the gas phase, the two modes belong to different symmetry species ($A_{1}$ and $B_{2}$, respectively), so are forbidden from mixing. In the solid state, the requirement is that the symmetry of the mode is for the entire primitive cell: it is not restricted to a single molecule, as is the case in the isolated molecule situation.^[^ ^38^ ^]^ Figure 5, bottom (left and right, resp.) show examples of modes that are nominally $\nu_{2}$ and $\nu_{6}$, respectively, it can be seen that both have molecules that are executing in‐plane ($\nu_{2}$‐type) and out‐of‐plane ($\nu_{6}$‐type) motions.

In contrast to the lower energy modes, both $\nu_{4}$ and $\nu_{1}$ show significant dispersion of ca. 50 ${\mathrm{cm}}^{-1}$, consistent with the width of $\nu_{4}$ seen experimentally.

## Conclusion

The crystallographic analysis of solid phosgene has led to the structural characterisation of a metastable polymorph, denoted as β‐phosgene, whose existence hitherto has only been inferred by DSC measurements. Although the structure of α‐phosgene has been known since 1952 and is readily obtained by crystallisation through simple slow cooling, quench cooling of liquid phosgene to 135 K allowed isolation of β‐phosgene as a phase‐pure microcrystalline powder. Structure solution and Rietveld refinement of high‐resolution powder neutron diffraction data revealed that β‐phosgene crystallises in the orthorhombic space group $Cmc2_{1}$ (a=10.244042(22) Å, b=6.280321(21) Å, c=5.46069(4) Å, Z=4) with the centroids forming the packing motif of the α‐U type. Hirshfeld surface analysis shows both polymorphs exhibiting C⋯O (dipole–dipole) interactions, but only the more stable α‐polymorphs also shows σ‐hole interactions trough O⋯Cl contacts.

Dispersion‐corrected hybrid DFT calculations accurately reproduce the experimental lattice parameters and reveal that the α‐phosgene is energetically favoured by 0.5 kJ ${\mathrm{mol}}^{-1}$ per formula unit. Gas‐phase cluster models identify a range of intermolecular interactions (C⋯O, Cl⋯O, and Cl⋯Cl contacts), whose interplay and magnitude is different for α‐ and β‐phosgene. The dipole–dipole interaction (dimer A, mainly C⋯O) is the largest contributing factor in α‐phosgene; a close second is dimer C with contacts between 3.5 and 3.8 Å. Despite being prominently visible in the Hirshfeld surface analysis, the σ‐hole interaction contributes only weakly to the overall energy. In β‐phosgene dimers A and B are close in energy and an allegedly cominant contributing factor of the C⋯O contact can only be interpreted from tilting of the molecules in the crystal structure and the associated Hirshfeld analysis. Consequently, there is no clear definitive difference between both polymorphs and more research on other related systems is currently under way.

At low wavenumbers, inelastic neutron scattering shows an unusual separation of external modes in translation and libration. This is due to the low vibrational dispersion seen in the calculated curves and the modes do not mix. Similarly, $\nu_{3}$, $\nu_{5}$, $\nu_{2}$, and $\nu_{6}$ also show very little dispersion thus leading to sharp bands in the INS spectrum. However, here, we observe an extensive mixing of $\nu_{2}$ and $\nu_{6}$, which is symmetry forbidden in the gas‐phase.

## Conflict of Interests

The authors declare no conflict of interest.

## Supporting information

Supporting Information

Supporting Information

## Data Availability

The data that support the findings of this study are available in the Supporting Information of this article.
