# Supplementary material for: Crystal Structures and Intermolecular Interactions in α‐ and β‐phosgene
Source: Angew Chem Int Ed Engl. 2025 Nov 14;65(2):e17323. doi: 10.1002/anie.202517323 (PMC12790384; doi:10.1002/anie.202517323)
Supplement: Supplementary file 1 — Supporting Information [file ANIE-65-e17323-s002.pdf]

Electronic Supporting Information (ESI)

Working Title

Sven Ringelband, Jonathan Pfeiffer, A. Dominic Fortes, Christopher M. Howard, Stewart F. Parker, Antti J. Karttunen, and Frank Tambornino*

Contents

1. Experimental section 2

1.1. General remarks 2

1.2. Single crystal X-ray diffraction 2

1.3. Neutron powder diffraction 3

1.4. Structure solution of β-phosgene 4

1.5. Details on Rietveld refinement of α- and β-phosgene 5

2. Additional pictures for phosgene 9

2.1. Data from single-crystal X-ray diffraction at 80 K α-phosgene 9

2.2. Data from neutron powder diffraction at 12 K α-phosgene 10

2.3. Additional pictures for β-phosgene from neutron powder diffraction at 135 K 11

3. Additional details on Hirshfeld surface analysis 13

3.1. α-phosgene at 100 K based on the single crystal X-ray structure 13

3.2. β-phosgene at 135 K based on the neutron powder diffraction data 14

4. Details on quantum chemical calculations with CASTEP and simulation of INS spectra 15

5. Details on quantum chemical calculations with CRYSTAL23 and TURBOMOLE 18

6. References 23

1. Experimental section

1.1. General remarks

For single crystal X-ray diffraction phosgene (GHC Gerling, Holz & Co., 1.85) was used as received. Triphosgene (Carbolution, 99%) was used as received. Copper phthalocyanine (Sigma-Alrich, triple-sublimed grade, >99.95% trace metals basis) was used as received.

For neutron powder diffraction, phosgene was synthesized from triphosgene according to literature.[1]

1.2. Single crystal X-ray diffraction

A small amount of phosgene was condensed into a glass capillary (diameter 0.3 mm, Lindemann Spezialglas) which was subsequently flame-sealed under vacuum. It was then centred on the goniometer of a StadiVari Diffractometer (Stoe & Cie GmbH, Darmstadt, Germany), equipped with an Oxford cryostream set to room temperature. Upon cooling at 10 K/min the sample rapidly solidified at ca. 120 K (−153.5 °C), which points to supercooling as the reported melting point is 155.15 K (−118 °C). During a test measurement polycrystallinity was observed. Hence, the sample was carefully and slowly heated until partial melting of the polycrystalline sample was visually observed. From here on, careful Ostwald-ripening with a sinusoidal temperature profile (±5 °C) was performed around the melting point, until the growth of a single crystal in the capillary was observed. Test measurements confirmed the growth of a single crystal in the tetragonal system with lattice parameters closely matching those of the reported crystal structure.[2] The specimen was cooled to 80 K before a full dataset was collected. Details for single crystal diffraction can be found in Table S 2.

1.3. Neutron powder diffraction

Phosgene was synthesized from triphosgene according to literature.[1] Subsequently, phosgene was condensed into a pre-cooled aluminium slab-geometry container (internal dimensions 18 x 23 mm perpendicular to the incident neutron beam and 10 mm deep parallel to the beam) held in place by steel-framed vanadium foil windows on the beam in/out faces that were sealed with indium wire. Exposed components of the cell were masked with Gd and Cd foils to prevent unwanted scattering from the Al and steel components of the slab can. Additionally, the cell was filled with dried glass-wool to prevent the formation of large single crystal areas or a sample with too strong preferred orientation. The temperature was controlled through a 30 mm cartridge heater which was inserted into one side of the aluminium frame of the sample holder slab with a RhFe resistance thermometer on the opposite side. The full assembly was the transferred into a closed-cycle refrigerator (CCR) held at 100 K.

Data were collected at the ISIS neutron spallation source at the High-Resolution Powder Diffractometer (HRPD) using the instrument's standard 30–130 ms time-of-flight (TOF) measurement window. Detector banks are positioned in backscattering geometry ($2\Theta = 158$–$176°$), at 90-degrees to the incident beam ($2\ \Theta = 80$–$100°$) and in forward scattering ($2\Theta = 28$–$32°$), providing *d*-spacing coverage in the 30-130 ms TOF window from 0.65–2.60, 0.85–3.90 and 2.3–10.2 Å, respectively. Diffraction data were time-focussed, normalised to the incident spectrum, corrected for instrument efficiency by reference to a V:Nb null-scattering standard, and corrected for the estimated sample absorption using Mantid[3] and then exported in a format suitable for analysis using standard Rietveld refinement codes. Measurements of an empty sample holder in a CCR were later subtracted in an effort to remove parasitic contributions from the sample environment equipment.

Initial measurements were performed in 10 K increments starting from 100 K down to 10 K. Longer integrations (5 h) suitable for both structure solution and Rietveld refinements were

collected at 100 K and 12 K. For the crystallization of the first metastable phase (phosgene-II or β-phosgene) the temperature profile from literature was applied.[4] From 165 K (−108.15 °C) the sample was first rapidly cooled (3.75 K min^{-1}) to 140 K (−133.15 °C). A supercooled melt was obtained, evident by the missing response of the heat of crystallization on the thermocouple and, more importantly, by the absence of sharp Bragg peaks. Thereafter, the sample was cooled very slowly (0.0167 K min^{-1}) to 135 K (−138.15 °C). During which time the data collection was continued. After 3 h 14 m, at a temperature of 136.8 K, the sample spontaneously crystallized, concomitant with a small spike (0.2 K, duration 90 s) in the observed temperature. Data for this metastable phase (phosgene-II, β-phosgene) was collected for 3 h 50 m to allow for indexing, structure solution and subsequent Rietveld refinement.

Several attempts were made to crystallize phosgene-III, which was reported in literature.[4] The temperature profile from literature was used (slowly cooling the liquid at 0.5 K min^{-1} to 130–135 K until a solid formed), however, in all experiments the stable form of phosgene-I (α-phosgene) was obtained. As stated in literature “Solid III is very unstable”, thus we do not rule out the existence of this phase.

1.4. Structure solution of β-phosgene

The powder neutron diffraction pattern was indexed with DICVOL06[5] as implemented in DASH 4.0.0.[6] The first 20 reflex positions with sharp profiles were selected for fitting and indexing resulted in an orthorhombic cell with a = 10.24807(29) Å, b = 6.28358(18) Å, c = 5.46339(22) Å, and a unit-cell volume V = 351.81 Å^3, commensurate with Z = 4; the figures of merit for this solution were M(20) = 90.0[7] and F(20) = 98.4(0.0026,79).[8] Systematic absences clearly indicated a C-centred orthorhombic cell; subsequent Pawley fitting narrowed the most likely options to either the non-centrosymmetric space group $Cmc2_1$ or the centrosymmetric space group *Cmce*. The structure solution was performed with TOPAS academic[9] with charge flipping methods.

1.5. Details on Rietveld refinement of α- and β-phosgene

All of the diffraction data were processed using the General Structure Analysis System (GSAS) with the Expgui graphical interface.[10,11] Significant contaminants, most likely from the sample environment, were identified in the initial powder data, these being copper and iron with relatively broad Bragg peaks. Due to the nature of the sample, there were no practical interventions possible to try and eliminate these parasitic phases and so all of the diffraction data were affected. Furthermore, subtraction of the empty cell + CCR failed to remove these features, and their origin remains mysterious. These phases also proved to be highly textured and so could not be well fitted by the Rietveld method using the tools available in GSAS for modelling preferred orientation. Consequently, the parasitic phases were treated by the LeBail method - “F(calc) Weighted” algorithm in GSAS – whilst the primary phosgene phases were treated by the Rietveld method.

Despite the application of an estimated absorption correction, it was still found necessary to refine an absorption coefficient in order to avoid the anisotropic displacement ellipsoid of the carbon atom in α-phosgene from turning non-positive definite at low temperature; even then, the resulting displacement ellipsoid adopted an extremely flattened form. Treating the carbon atom in α-phosgene with isotropic ADPs and the remaining atoms with anisotropic ADPs ultimately yielded a satisfactory fit of the powder data.

β-phosgene was found to exhibit a significant degree of preferred orientation, likely a reflection of the diminishing capacity of the silica wool to nucleate a good powder through more than one cycle of melting and recrystallisation. The preferred orientation in β-phosgene was treated with a 4th order spherical harmonic model in GSAS, yielding a texture index of 1.7848. All atoms were refined with anisotropic ADPs.

The completed refinements of the α-phosgene and β-phosgene structures were exported as Crystallographic Information Files (CIFs) for deposition with the CCDC, alongside CheckCIF reports from the IUCr's CIF structure refinement validation service (https://checkcif.iucr.org).

Table S 1: Selected single crystal X-ray data collection and refinement parameters for α-phosgene.

	α-phosgene
Formula	$COCl_2$
CCDC	2478734
F. w. / g mol^{-1}	98.91
Crystal system	tetragonal
Space group	$I4_1/a$
a / Å	15.6974(4)
b / Å	15.6974(4)
c / Å	5.6780(2)
α / °	90
β / °	90
γ / °	90
V / Å^3	1399.11(9)
Z	16
Radiation, λ / Å	1.54186
Temp / K	80
ρ_{calc} / g cm^{-3}	1.878
μ / mm^{-1}	14.722
Reflections collected	21383
Ind. Reflns. / Ind. Reflns gt	658 / 645
Parameters	37
R_{int} / $R_{(\sigma)}$ / %	4.81 / 0.95
$R1/wR2$,[a] $I \geq 2\sigma I$ / %	3.47 / 9.86
$R1/wR2$,[a] all data / %	3.52 / 9.94
GOF	1.120
Twin law	n/a
BASF	n/a
Flack parameter	n/a

[a] $R1 = [\Sigma||F_o| - |F_c||]/\Sigma|F_o|$; $wR2 = \{[\Sigma w[(F_o)^2 - (F_c)^2]^2]/[\Sigma w(F_o^2)^2\}^{1/2}$; $w = [\sigma^2(F_o)^2 + (AP)^2 + BP]^{-1}$, where $P = [(F_o)^2 + 2(F_c)^2]/3$ and the A and B values are 0.078500 and 0.491300 for α-phosgene.

Table S 2: Selected neutron powder data collection and refinement parameters for phosgene.

	α-phosgene (12 K)	α-phosgene (100 K)	β-phosgene (135 K)
Formula	$COCl_2$	$COCl_2$	$COCl_2$
CCDC	2478728	2478731	2478725
Fw / g mol^{-1}	98.91	98.91	98.91
Crystal system	Tetragonal	Tetragonal	Orthorhombic
Space group	$I4_1/a$	$I4_1/a$	$Cmc2_1$
a / Å	15.58245(4)	15.71613(4)	10.244042(22)
b / Å	15.58245(4)	15.71613(4)	6.280321(21)
c / Å	5.655737(25)	5.684833(25)	5.46069(4)
α / °	90	90	90
β / °	90	90	90
γ / °	90	90	90
V / Å^3	1373.285(8)	1404.135(7)	351.319(2)
Z	16	16	4
Radiation, λ / Å	0.5 – 8.2	0.5 – 8.2	0.5 – 8.2
Temp / K	12	100	135
ρ_{calc} / g cm^{-3}	1.91367	1.87162	1.87011
R_{wp} / %	0.0503	0.0458	0.0549
R_p / %	0.0627	0.0603	0.0659
GoF	1.94	1.63	1.77
Refined Parameters	36	48	31
Background Parameters	12	12	12
Profile Parameters[a]	6	6	12

[a] Profile parameters include spherical harmonics function up to order 4 to model preferred orientation.

2. Additional pictures for phosgene

2.1. Data from single-crystal X-ray diffraction at 80 K α-phosgene

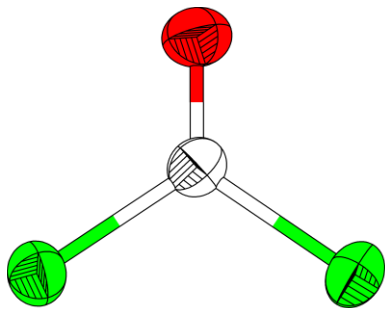

Figure S 1: Molecular structure of phosgene in α-phosgene as deduced from single crystal X-ray diffraction. Displacement ellipsoids are shown with 75% probability level at 80 K. Colour code: O red, C white, Cl green.

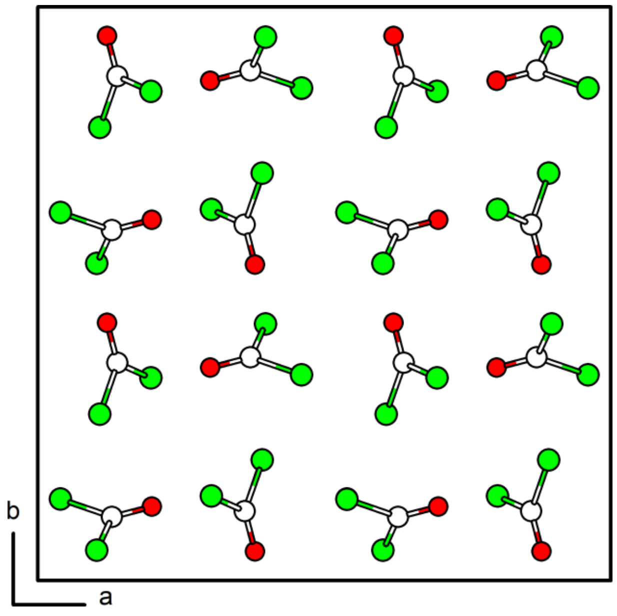

Figure S 2: Crystal structure of α-phosgene viewed along [001] as deduced from single crystal X-ray diffraction at 80 K. Atoms drawn with arbitrary radii. Colour code: O red, C white, Cl green.

2.2. Data from neutron powder diffraction at 12 K α-phosgene

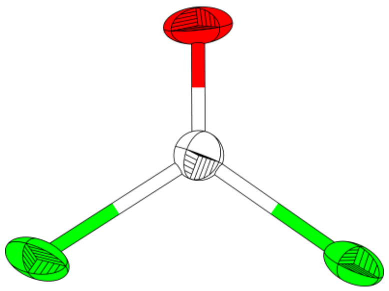

Figure S 3: Molecular structure of phosgene in α-phosgene as deduced from neutron powder diffraction. Displacement ellipsoids are shown with 90% probability level at 12 K. Colour code: O red, C white, Cl green.

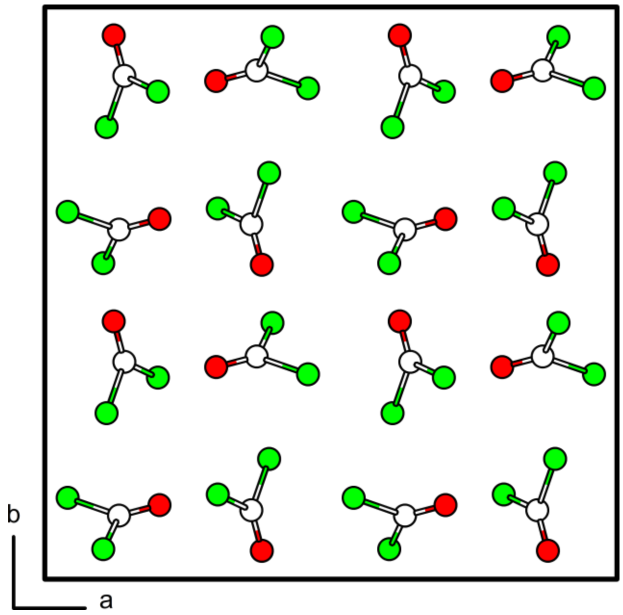

Figure S 4: Crystal structure of α-phosgene viewed along [001] as deduced from neutron powder diffraction at 12 K. Atoms drawn with arbitrary radii. Colour code: O red, C white, Cl green.

2.3. Additional pictures for β-phosgene from neutron powder diffraction at 135 K

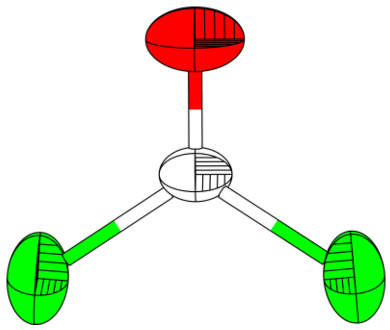

Figure S 5: Molecular structure of phosgene in β-phosgene as deduced from neutron powder diffraction. Displacement ellipsoids are shown with 75% probability level at 135 K. Colour code: O red, C white, Cl green.

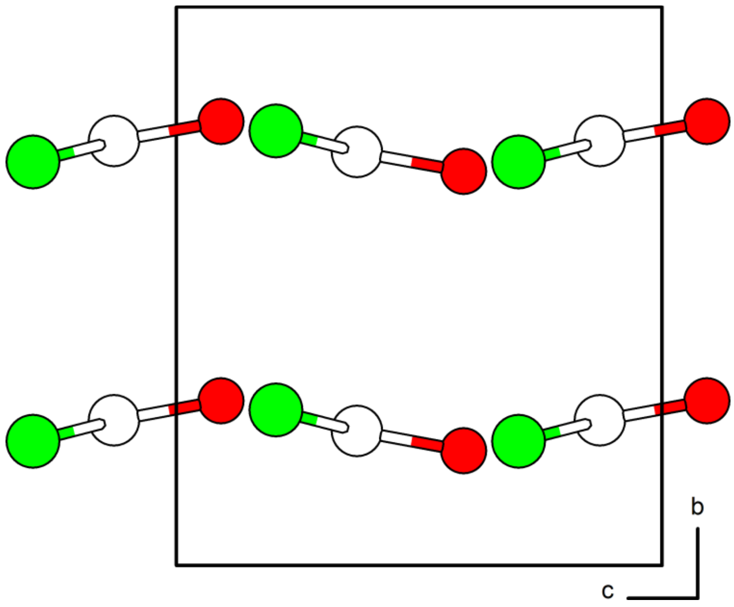

Figure S 6: Crystal structure of β-phosgene viewed along [100]. Atoms are drawn with arbitrary radii. Colour code: O red, C white, Cl green.

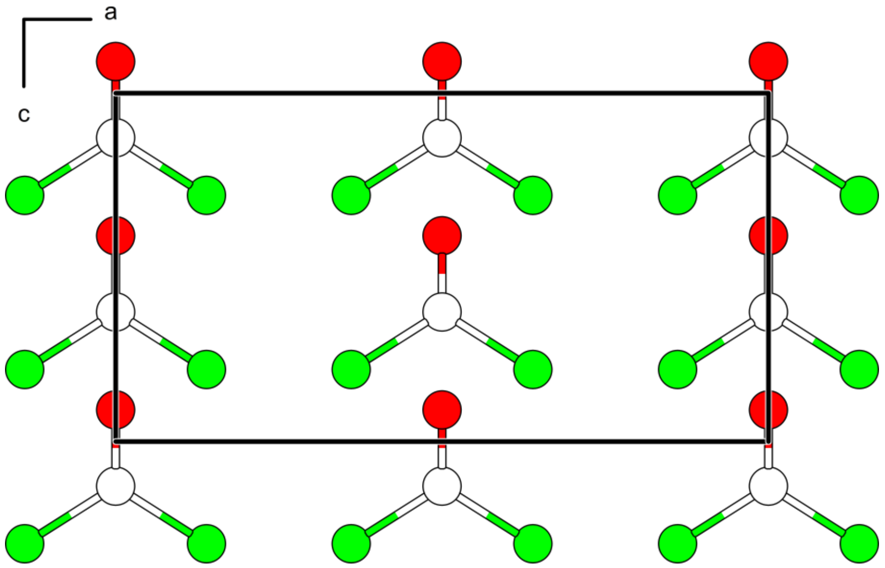

Figure S 7: Crystal structure of β-phosgene viewed along [010]. Atoms are drawn with arbitrary radii. Colour code: O red, C white, Cl green.

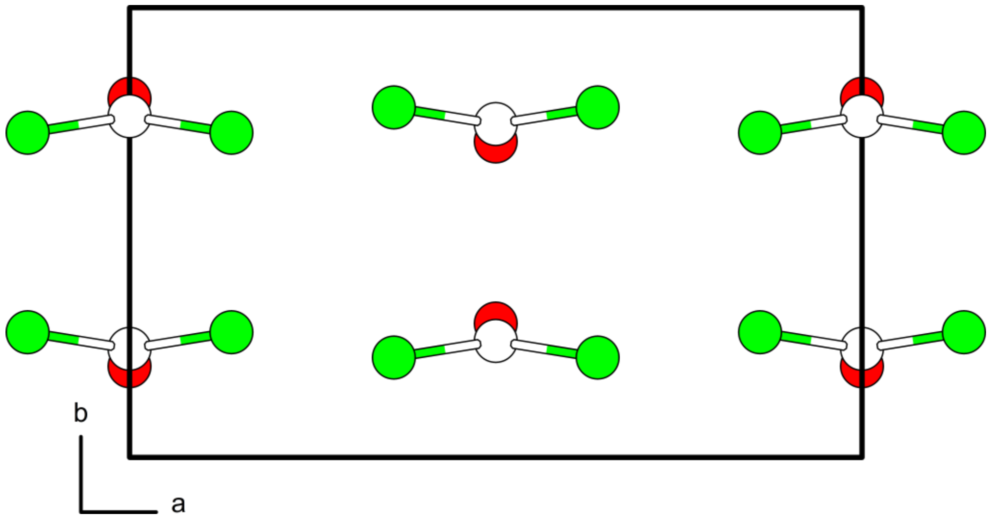

Figure S 8: Crystal structure of β-phosgene viewed along [001]. Atoms are drawn with arbitrary radii. Colour code: O red, C white, Cl green.

3. Additional details on Hirshfeld surface analysis

3.1. α-phosgene at 100 K based on the single crystal X-ray structure

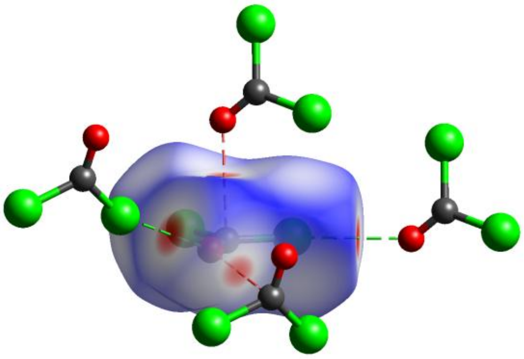

Figure S 9: Hirshfeld surface of α-phosgene shown with neighboring molecules. Red areas indicate short contacts. Red dashed lines highlight C···O short contacts, green dashed lines highlight Cl···O short contacts. Colour code: O red, C grey, Cl green.

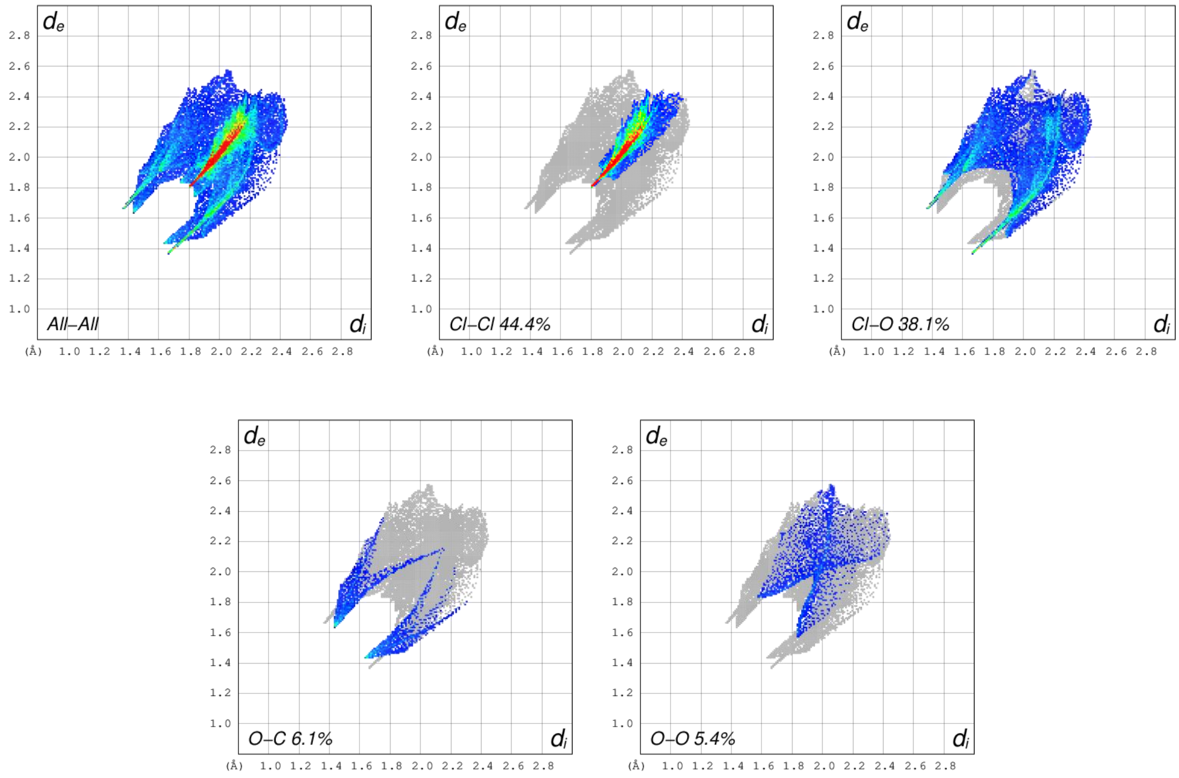

Figure S 10: Fingerprint plots for α-phosgene mapped from the Hirshfeld surface. Surface contacts and overall quantity are provided in each graph.

3.2. β-phosgene at 135 K based on the neutron powder diffraction data

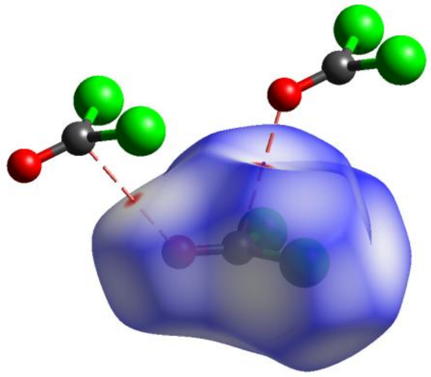

Figure S 11: Hirshfeld surface of β-phosgene shown with neighboring molecules. Red areas indicate short contacts. Red dashed lines highlight C⋯O short contacts. Colour code: O red, C grey, Cl green.

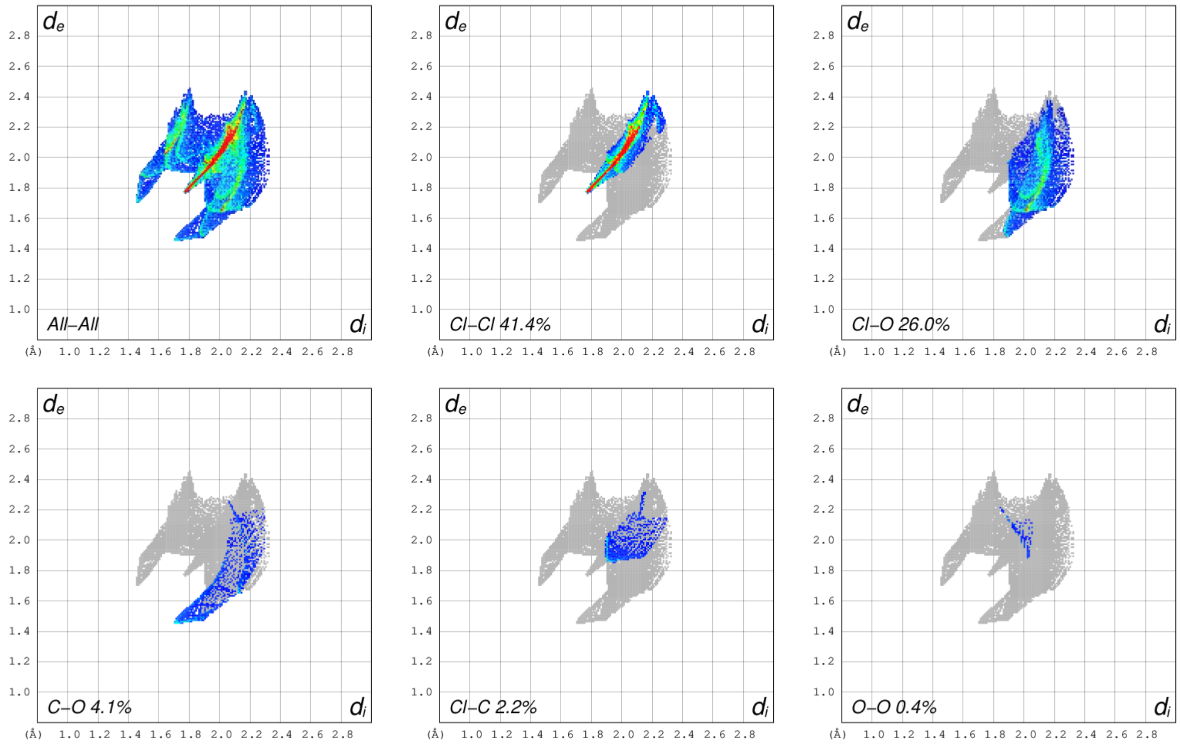

Figure S 12: Fingerprint plots for β-phosgene mapped from the Hirshfeld surface. Surface contacts and overall quantity are provided in each graph.

4. Details on quantum chemical calculations with CASTEP and simulation of INS spectra

The INS spectra were recorded using TOSCA.[12,13] Phosgene was prepared as previously described[1] and loaded into an In wire sealed Al can. The sample was then cooled to ~20 K and the spectrum recorded for ~17 hours.

Dispersion corrected periodic DFT calculations of the crystalline structure were carried out using the plane-wave pseudopotential method implemented in the CASTEP code.[14] Exchange and correlation were approximated using the Perdew-Burke-Ernzerhof (PBE) functional,[15] with the Tkatchenko-Scheffler (TS) dispersion correction scheme[16] within the generalized gradient approximation (GGA). Norm-conserving pseudopotentials were generated using the kinetic-energy optimised method[17] with a plane-wave cut-off energy of 1020 eV. Brillouin-zone sampling of electronic states was performed on an 8×8×4 Monkhorst-Pack grid (104 k-points). The equilibrium structure, an essential prerequisite for lattice dynamics calculations was obtained by Broyden-Fletcher-Goldfarb-Shanno (BFGS) geometry optimization after which the residual forces were converged to zero within ±0.0012 eV $Å^{-1}$. Phonon frequencies were obtained by diagonalization of dynamical matrices computed using density-functional perturbation theory.[18] In addition to the direct evaluation of frequencies and intensities at zero wavevector, phonon dispersion was also calculated along high symmetry directions throughout the Brillouin zone. For this purpose, dynamical matrices were computed on a regular grid of wavevectors throughout the Brillouin zone and Fourier interpolation was used to extend the computed grid to the desired fine set of points along the high-symmetry paths.[19] The calculated INS spectrum was generated from the atomic displacements in each mode that are part of the CASTEP output, using AbINS.[20]

Table S 3: Ab initio transition energies, infrared intensities and assignments for α-phosgene.

Transition energy / cm^{-1}	Irreducible representation[a]	Infrared intensity / km mol^{-1}	Infrared allowed?	Raman allowed?	Average[b] / cm^{-1}	Range[c] / cm^{-1}	Assignment
0	Au	0,00	N	N	23	44	Acoustic translation
0	Eu	0,00	N	N			Acoustic translation
0	Eu	0,00	N	N			Acoustic translation
28	Bg	0,00	N	Y			Optic translation
32	Bu	0,00	N	N			Optic translation
38	Ag	0,00	N	Y			Optic translation
44	Eg	0,00	N	Y			Optic translation
44	Eg	0,00	N	Y			Optic translation
49	Eu	1,65	Y	N	54	8	Optic translation
49	Eu	1,65	Y	N			Optic translation
54	Au	2,59	Y	N			Optic translation
54	Bg	0,00	N	Y			Optic translation
54	Ag	0,00	N	Y			Optic translation
56	Eg	0,00	N	Y			Optic translation
56	Eg	0,00	N	Y			Optic translation
57	Bg	0,00	N	Y			Optic translation
57	Bu	0,00	N	N			Optic translation
63	Ag	0,00	N	Y	66	13	Optic translation
65	Bu	0,00	N	N			Optic translation
67	Eu	3,54	Y	N			Optic translation
67	Eu	3,54	Y	N			Optic translation
69	Eg	0,00	N	Y			Optic translation
69	Eg	0,00	N	Y			Optic translation
70	Au	1,20	Y	N			Optic translation
76	Eg	0,00	N	Y	81	11	Libration (axis perp to molecular plane)
76	Eg	0,00	N	Y			Libration (axis perp to molecular plane)
80	Au	8,55	Y	N			Libration (axis perp to molecular plane)
80	Ag	0,00	N	Y			Libration (axis perp to molecular plane)
80	Eu	0,94	Y	N			Libration (axis perp to molecular plane)
80	Eu	0,94	Y	N			Libration (axis perp to molecular plane)
86	Bu	0,00	N	N			Libration (axis perp to molecular plane)
86	Eg	0,00	N	Y	95	17	Libration (rot about C=O axis)
86	Eg	0,00	N	Y			Libration (rot about C=O axis)
87	Bg	0,00	N	Y			Libration (rot about C=O axis)
92	Au	0,61	Y	N			Libration (rot about C=O axis)
96	Eu	1,41	Y	N			Libration (rot about C=O axis)
96	Eu	1,41	Y	N			Libration (rot about C=O axis)
97	Ag	0,00	N	Y			Libration (rot about C=O axis)
99	Eg	0,00	N	Y	104	9	Libration (rot about Cl-Cl)
99	Eg	0,00	N	Y			Libration (rot about Cl-Cl)
102	Bg	0,00	N	Y			Libration (rot about Cl-Cl)
103	Eu	0,35	Y	N			Libration (rot about Cl-Cl)
103	Eu	0,35	Y	N			Libration (rot about Cl-Cl)
103	Bu	0,00	N	N			Libration (rot about Cl-Cl)
107	Bg	0,00	N	Y			Libration (rot about Cl-Cl)
108	Bu	0,00	N	N			Libration (rot about Cl-Cl)
108	Ag	0,00	N	Y			Libration (rot about Cl-Cl)
108	Au	13,61	Y	N			Libration (rot about Cl-Cl)
297	Au	0,00	Y	N	298	4	In-phase Cl–C–Cl in-plane bend (A1)
297	Eu	4,95	Y	N			In-phase Cl–C–Cl in-plane bend (A1)
297	Eu	4,95	Y	N			In-phase Cl–C–Cl in-plane bend (A1)
298	Eg	0,00	N	Y			In-phase Cl–C–Cl in-plane bend (A1)
298	Eg	0,00	N	Y			In-phase Cl–C–Cl in-plane bend (A1)
298	Bg	0,00	N	Y			In-phase Cl–C–Cl in-plane bend (A1)
300	Bu	0,00	N	N			In-phase Cl–C–Cl in-plane bend (A1)
301	Ag	0,00	N	Y			In-phase Cl–C–Cl in-plane bend (A1)
432	Bg	0,00	N	Y	435	6	Out-of-phase Cl–C–Cl in-plane bend (B1)
434	Bu	0,00	N	N			Out-of-phase Cl–C–Cl in-plane bend (B1)
434	Eg	0,00	N	Y			Out-of-phase Cl–C–Cl in-plane bend (B1)
434	Eg	0,00	N	Y			Out-of-phase Cl–C–Cl in-plane bend (B1)
435	Eu	5,93	Y	N			Out-of-phase Cl–C–Cl in-plane bend (B1)
435	Eu	5,93	Y	N			Out-of-phase Cl–C–Cl in-plane bend (B1)
437	Au	1,93	Y	N			Out-of-phase Cl–C–Cl in-plane bend (B1)
438	Ag	0,00	N	Y			Out-of-phase Cl–C–Cl in-plane bend (B1)
560	Eu	178,63	Y	N	563	6	In-phase C–Cl stretch (A1)
560	Eu	178,63	Y	N			In-phase C–Cl stretch (A1)
560	Bg	0,00	N	Y			In-phase C–Cl stretch (A1)

562	Bu	0,00	N	N			In-phase C–Cl stretch (A1)
565	Eg	0,00	N	Y			In-phase C–Cl stretch (A1)
565	Eg	0,00	N	Y			In-phase C–Cl stretch (A1)
565	Ag	0,00	N	Y			In-phase C–Cl stretch (A1)
566	Au	16,49	Y	N			In-phase C–Cl stretch (A1)
569	Eg	0,00	N	Y	570	3	Out-of-plane bend (B2)
569	Eg	0,00	N	Y			Out-of-plane bend (B2)
569	Bu	0,00	N	N			Out-of-plane bend (B2)
569	Eu	3,01	Y	N			Out-of-plane bend (B2)
569	Eu	3,01	Y	N			Out-of-plane bend (B2)
570	Au	8,11	Y	N			Out-of-plane bend (B2)
570	Ag	0,00	N	Y			Out-of-plane bend (B2)
572	Bg	0,00	N	Y			Out-of-plane bend (B2)
774	Eg	0,00	N	Y	790	45	Out-of-phase C–Cl stretch (B1)
774	Eg	0,00	N	Y			Out-of-phase C–Cl stretch (B1)
777	Bg	0,00	N	Y			Out-of-phase C–Cl stretch (B1)
788	Eu	1183,63	Y	N			Out-of-phase C–Cl stretch (B1)
788	Eu	1183,63	Y	N			Out-of-phase C–Cl stretch (B1)
796	Ag	0,00	N	Y			Out-of-phase C–Cl stretch (B1)
805	Bu	0,00	N	N			Out-of-phase C–Cl stretch (B1)
819	Au	3218,36	Y	N			Out-of-phase C–Cl stretch (B1)
1781	Ag	0,00	N	Y	1788	15	C=O stretch (A1)
1782	Eu	2578,79	Y	N			C=O stretch (A1)
1782	Eu	2578,79	Y	N			C=O stretch (A1)
1783	Au	383,74	Y	N			C=O stretch (A1)
1788	Bu	0,00	N	N			C=O stretch (A1)
1794	Bg	0,00	N	Y			C=O stretch (A1)
1796	Eg	0,00	N	Y			C=O stretch (A1)
1796	Eg	0,00	N	Y			C=O stretch (A1)

[a] Irreducible representation for space group.
[b] Average of the factor group components.
[c] Difference between the highest and lowest energy of the factor group components.

5. Details on quantum chemical calculations with CRYSTAL23 and TURBOMOLE

Solid-state calculations on the energetics and thermodynamics of the phosgene polymorphs were carried out with the CRYSTAL23 program package.[21] PBE0 hybrid density functional method (PBE exchange-correlation and 25% exact HF exchange) with Grimme's D3 dispersion correction was used (D3 with Becke-Johnson damping and three-body ABC correction).[22–26] As the phosgene polymorphs are molecular crystals, molecular def2-TZVP polarized triple-ζ-valence basis could be applied for all atoms without any modifications.[27] Crystal structures were fully optimized within the symmetry constrains of the space group. The reciprocal space was sampled with a 2 × 2 × 2 and 4 × 4 × 4 Monkhorst-Pack-type *k*-point grid for the *α* and *β* polymorph, respectively.[28] The optimized crystal structures are available below in CIF format. Tight tolerances (TOLINTEG) of 8, 8, 8, 8, and 16 were used for the evaluation of the Coulomb and exchange integrals. Default optimization criteria and DFT integration grids of CRYSTAL23 were applied. Harmonic frequency calculations were carried out with the finite-displacement approach implemented in CRYSTAL.[29] Harmonic frequency calculations at the Γ point showed both studied polymorphs to be true local minima with no imaginary frequencies. Gibbs free energies were obtained for β-phosgene using a 2 × 2 × 1 phonon supercell that has the same number of atoms as α-phosgene in its primitive cell.

Molecular calculations on gas-phase model systems were carried out with the TURBOMOLE code (version 7.8).[30] Explicitly correlated Coupled Cluster Singles and Doubles with perturbative triples method, CCSD(F12*)(T*), and cc-pVTZ-F12 triple-ζ-valence basis set were applied.[31,32] Default freezing criteria for the core orbitals were applied. Resolution-of-the-identity approximation was used to speed up the Coupled Cluster calculations carried out with ccsdf12 module.[33] The coordinates of the studied molecular model systems are available below.

Optimized crystal structures in CIF format

α-phosgene (dispersion-corrected DFT-PBE0)

```
data_findsym-output
_audit_creation_method FINDSYM

_cell_length_a    15.7220257586
_cell_length_b    15.7220257586
_cell_length_c    5.7321904173
_cell_angle_alpha 90.0000000000
_cell_angle_beta  90.0000000000
_cell_angle_gamma 90.0000000000
_cell_volume      1416.8948303001

_symmetry_space_group_name_H-M "I 41/a (origin choice 2)"
_symmetry_Int_Tables_number 88
_space_group.reference_setting '088:-I 4ad'
_space_group.transform_Pp_abc a,b,c;0,0,0

loop_
_space_group_symop_id
_space_group_symop_operation_xyz
1 x,y,z
2 -x,-y+1/2,z
3 -y+3/4,x+1/4,z+1/4
4 y+1/4,-x+1/4,z+1/4
5 -x,-y,-z
6 x,y+1/2,-z
7 y+1/4,-x+3/4,-z+3/4
8 -y+3/4,x+3/4,-z+3/4
9 x+1/2,y+1/2,z+1/2
10 -x+1/2,-y,z+1/2
11 -y+1/4,x+3/4,z+3/4
12 y+3/4,-x+3/4,z+3/4
13 -x+1/2,-y+1/2,-z+1/2
14 x+1/2,y,-z+1/2
15 y+3/4,-x+1/4,-z+1/4
16 -y+1/4,x+1/4,-z+1/4

loop_
_atom_site_label
_atom_site_type_symbol
_atom_site_symmetry_multiplicity
_atom_site_Wyckoff_label
_atom_site_fract_x
_atom_site_fract_y
_atom_site_fract_z
_atom_site_occupancy
_atom_site_fract_symmform
Cl1 Cl  16 f 0.19693 0.85239 0.01118 1.00000 Dx,Dy,Dz
Cl2 Cl  16 f 0.60715 0.29177 0.10769 1.00000 Dx,Dy,Dz
O1  O   16 f 0.62144 0.45014 0.21664 1.00000 Dx,Dy,Dz
C1  C   16 f 0.63892 0.37988 0.26823 1.00000 Dx,Dy,Dz

# end of cif
```

β-phosgene (dispersion-corrected DFT-PBE0)

```
data_findsym-output
_audit_creation_method FINDSYM

_cell_length_a    10.1587209084
_cell_length_b    6.1642564895
_cell_length_c    5.5257113600
_cell_angle_alpha 90.0000000000
_cell_angle_beta  90.0000000000
_cell_angle_gamma 90.0000000000
_cell_volume      346.0253571478

_symmetry_space_group_name_H-M "C m c 21"
_symmetry_Int_Tables_number 36
_space_group.reference_setting '036:C 2c -2'
_space_group.transform_Pp_abc a,b,c;0,0,0

loop_
_space_group_symop_id
_space_group_symop_operation_xyz
1 x,y,z
2 -x,-y,z+1/2
3 -x,y,z
4 x,-y,z+1/2
5 x+1/2,y+1/2,z
6 -x+1/2,-y+1/2,z+1/2
7 -x+1/2,y+1/2,z
8 x+1/2,-y+1/2,z+1/2

loop_
_atom_site_label
_atom_site_type_symbol
_atom_site_symmetry_multiplicity
_atom_site_Wyckoff_label
_atom_site_fract_x
_atom_site_fract_y
_atom_site_fract_z
_atom_site_occupancy
_atom_site_fract_symmform
Cl1 Cl   8 b 0.35876 0.22306 0.79420  1.00000 Dx,Dy,Dz
C1  C    4 a 0.00000 0.24759 0.12306  1.00000 0,Dy,Dz
O1  O    4 a 0.00000 0.21244 -0.08626 1.00000 0,Dy,Dz

# end of cif
```

Optimized molecular dimers in XYZ format

α-phosgene dimer A

```
C     5.8190488   -1.7464696    0.1044822
O     4.7143835   -2.0212406   -0.1912058
Cl    6.2512253   -0.8342844    1.4971224
Cl    7.2043488   -2.2458601   -0.8157515
O     5.9517471    0.7838770   -1.6242534
C     5.6769760    1.8885424   -1.3285654
Cl    4.7647909    2.3207188    0.0640748
Cl    6.1763665    3.2738424   -2.2487990
```

α-phosgene dimer B

```
C     5.8190488   -1.7464696    0.1044822
O     4.7143835   -2.0212406   -0.1912058
Cl    6.2512253   -0.8342844    1.4971224
Cl    7.2043488   -2.2458601   -0.8157515
Cl    3.0962220   -2.3207188   -2.8020203
C     2.1840369   -1.8885424   -4.1946606
O     1.9092658   -0.7838770   -4.4903485
Cl    1.6846464   -3.2738424   -5.1148942
```

α-phosgene dimer C

```
C     5.8190488   -1.7464696    0.1044822
O     4.7143835   -2.0212406   -0.1912058
Cl    6.2512253   -0.8342844    1.4971224
Cl    7.2043488   -2.2458601   -0.8157515
Cl    6.1763665   -4.5871705    2.2487990
C     5.6769760   -5.9724705    1.3285654
O     5.9517471   -7.0771359    1.6242534
Cl    4.7647909   -5.5402941   -0.0640748
```

α-phosgene dimer D

```
Cl   -0.6566641    5.6151528    2.0503437
C    -2.0419641    6.1145433    2.9705773
O    -3.1466294    5.8397723    2.6748894
Cl   -1.6097876    7.0267285    4.3632176
Cl    0.6566641    2.2458601    2.0503437
C     2.0419641    1.7464696    2.9705773
O     3.1466294    2.0212406    2.6748894
Cl    1.6097876    0.8342844    4.3632176
```

α-phosgene dimer E

```
Cl   -0.6566641    5.6151528    2.0503437
C    -2.0419641    6.1145433    2.9705773
O    -3.1466294    5.8397723    2.6748894
Cl   -1.6097876    7.0267285    4.3632176
Cl    1.6846464    4.5871705   -0.6172961
C     2.1840369    5.9724705   -1.5375297
O     1.9092658    7.0771359   -1.2418418
Cl    3.0962220    5.5402941   -2.9301700
```

β-phosgene dimer A

```
C     0.0000000    1.5261874   -2.0828366
O     0.0000000    1.3095221   -3.2395273
Cl    1.4347930    1.7071314   -1.1371921
Cl   -1.4347930    1.7071314   -1.1371921
O     0.0000000   -1.3095221   -0.4766716
C     0.0000000   -1.5261874    0.6800191
Cl   -1.4347930   -1.7071314    1.6256636
Cl    1.4347930   -1.7071314    1.6256636
```

β-phosgene dimer B

Cl	1.4347930	1.7071314	-1.1371921
C	0.0000000	1.5261874	-2.0828366
O	0.0000000	1.3095221	-3.2395273
Cl	-1.4347930	1.7071314	-1.1371921
O	0.0000000	4.8547343	-0.4766716
C	0.0000000	4.6380691	0.6800191
Cl	-1.4347930	4.4571251	1.6256636
Cl	1.4347930	4.4571251	1.6256636

β -phosgene dimer C

Cl	1.4347930	1.7071314	-1.1371921
C	0.0000000	1.5261874	-2.0828366
O	0.0000000	1.3095221	-3.2395273
Cl	-1.4347930	1.7071314	-1.1371921
Cl	3.6445675	1.3749969	1.6256636
C	5.0793605	1.5559408	0.6800191
O	5.0793605	1.7726061	-0.4766716
Cl	6.5141535	1.3749969	1.6256636

6. References

[1] H. Eckert, J. Auerweck, *Org. Process Res. Dev.* **2010**, *14*, 1501–1505.
[2] B. Zaslow, M. Atoji, W. N. Lipscomb, *Acta Crystallogr.* **1952**, *5*, 833–837.
[3] O. Arnold, J. C. Bilheux, J. M. Borreguero, A. Buts, S. I. Campbell, L. Chapon, M. Doucet, N. Draper, R. Ferraz Leal, M. A. Gigg, V. E. Lynch, A. Markvardsen, D. J. Mikkelson, R. L. Mikkelson, R. Miller, K. Palmen, P. Parker, G. Passos, T. G. Perring, P. F. Peterson, S. Ren, M. A. Reuter, A. T. Savici, J. W. Taylor, R. J. Taylor, R. Tolchenov, W. Zhou, J. Zikovsky, *Nuclear Inst. and Methods in Physics Research A* **2014**, *764*, 156–166.
[4] W. F. Giauque, J. B. Ott, *J. Am. Chem. Soc.* **1960**, *82*, 2689–2695.
[5] D. Louër, A. Boultif, *Z. Kristallogr. Suppl.* **2007**, *26*, 191–196.
[6] W. I. F. David, K. Shankland, J. Van De Streek, E. Pidcock, W. D. S. Motherwell, J. C. Cole, *J. Appl. Crystallogr.* **2006**, *39*, 910–915.
[7] P. M. De Wolff, *J. Appl. Crystallogr.* **1968**, *1*, 108–113.
[8] G. S. Smith, R. L. Snyder, *J. Appl. Crystallogr.* **1979**, *12*, 60–65.
[9] A. A. Coelho, *J. Appl. Crystallogr.* **2018**, *51*, 210–218.
[10] A. C. Larson, R. B. Von Dreele, **2000**.
[11] B. H. Toby, *J. Appl. Crystallogr.* **2001**, *34*, 210–213.
[12] R. S. Pinna, S. Rudić, S. F. Parker, J. Armstrong, M. Zanetti, G. Škoro, S. P. Waller, D. Zacek, C. A. Smith, M. J. Capstick, D. J. McPhail, D. E. Pooley, G. D. Howells, G. Gorini, F. Fernandez-Alonso, *Nuclear Inst. and Methods in Physics Research A* **2018**, *896*, 68–74.
[13] S. F. Parker, F. Fernandez-Alonso, A. J. Ramirez-Cuesta, J. Tomkinson, S. Rudic, R. S. Pinna, G. Gorini, J. Fernández Castañon, *J. Phys.: Conf. Ser.* **2014**, *554*, 012003.
[14] S. J. Clark, M. D. Segall, C. J. Pickard, P. J. Hasnip, M. I. J. Probert, K. Refson, M. C. Payne, *Z. Kristallogr. - Cryst. Mater.* **2005**, *220*, 567–570.
[15] J. P. Perdew, K. Burke, M. Ernzerhof, *Phys. Rev. Lett.* **1997**, *78*, 1396–1396.
[16] A. Tkatchenko, M. Scheffler, *Phys. Rev. Lett.* **2009**, *102*, 073005.
[17] A. M. Rappe, K. M. Rabe, E. Kaxiras, J. D. Joannopoulos, *Phys. Rev. B* **1990**, *41*, 1227–1230.
[18] K. Refson, P. R. Tulip, S. J. Clark, *Phys. Rev. B* **2006**, *73*, 155114.
[19] X. Gonze, J.-C. Charlier, D. C. Allan, M. P. Teter, *Phys. Rev. B* **1994**, *50*, 13035–13038.
[20] K. Dymkowski, S. F. Parker, F. Fernandez-Alonso, S. Mukhopadhyay, *Physica B: Condensed Matter* **2018**, *551*, 443–448.
[21] A. Erba, J. K. Desmarais, S. Casassa, B. Civalleri, L. Donà, I. J. Bush, B. Searle, L. Maschio, L. Edith-Daga, A. Cossard, C. Ribaldone, E. Ascrizzi, N. L. Marana, J.-P. Flament, B. Kirtman, *J. Chem. Theory Comput.* **2023**, *19*, 6891–6932.
[22] J. P. Perdew, K. Burke, M. Ernzerhof, *Phys. Rev. Lett.* **1996**, *77*, 3865–3868.
[23] C. Adamo, V. Barone, *J. Chem. Phys.* **1999**, *110*, 6158–6170.
[24] T. Risthaus, S. Grimme, *J. Chem. Theory Comput.* **2013**, *9*, 1580–1591.
[25] S. Grimme, J. Antony, S. Ehrlich, H. Krieg, *J. Chem. Phys.* **2010**, *132*, 154104.
[26] S. Grimme, S. Ehrlich, L. Goerigk, *J Comput Chem* **2011**, *32*, 1456–1465.
[27] F. Weigend, R. Ahlrichs, *Phys. Chem. Chem. Phys.* **2005**, *7*, 3297.
[28] H. J. Monkhorst, J. D. Pack, *Phys. Rev. B* **1976**, *13*, 5188–5192.
[29] F. Pascale, C. M. Zicovich-Wilson, F. López Gejo, B. Civalleri, R. Orlando, R. Dovesi, *J. Comput. Chem.* **2004**, *25*, 888–897.
[30] R. Ahlrichs, M. Bär, M. Häser, H. Horn, C. Kölmel, *Chem. Phys. Lett.* **1989**, *162*, 165–169.
[31] R. A. Bachorz, F. A. Bischoff, A. Glöß, C. Hättig, S. Höfener, W. Klopper, D. P. Tew, *J. Comput. Chem.* **2011**, *32*, 2492–2513.

[32] K. A. Peterson, T. B. Adler, H.-J. Werner, *J. Chem. Phys.* **2008**, *128*, DOI 10.1063/1.2831537.
[33] C. Hättig, D. P. Tew, A. Köhn, *J. Chem. Phys.* **2010**, *132*, DOI 10.1063/1.3442368.
